# A Review of Microinjection Moulding of Polymeric Micro Devices

**DOI:** 10.3390/mi13091530

**Published:** 2022-09-16

**Authors:** Honggang Zhang, Haibin Liu, Nan Zhang

**Affiliations:** 1 Faculty of Materials and Manufacturing, Beijing University of Technology, Beijing 100124, China; 2 Centre of Micro/Nano Manufacturing Technology (MNMT-Dublin), School of Mechanical & Materials Engineering, University College Dublin, Belfield, D04 V1W8 Dublin, Ireland

**Keywords:** microinjection moulding, polymer devices, micro/nano structures

## Abstract

Polymeric micro devices are gaining huge market potential in broad areas of medical devices, diagnostic devices, drug delivery, and optical applications. Current research is focusing on developing functional polymeric micro devices on a mass-production scale. Microinjection moulding is a promising technique suitable for fabricating polymeric micro devices. This review aims to summarise the primary achievements that have been achieved in various aspects of microinjection moulding of polymer micro devices, consisting of micro parts and micro surface structures. The relationships of the machine, process, rheology, tooling, micro/nanoscale replication, morphology, properties, and typical applications are reviewed in detail. Finally, a conclusion and challenges are highlighted.

## 1. Introduction

**Figure 1:**
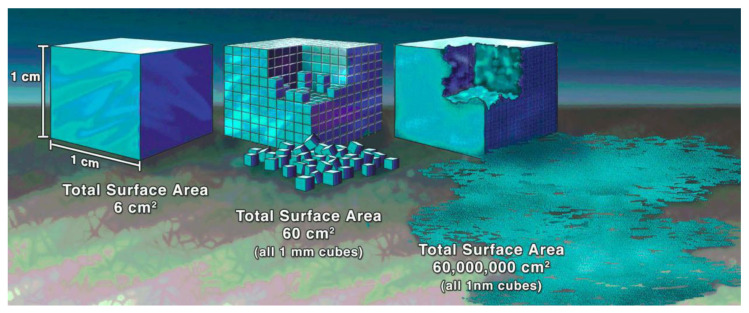
Comparison of surface areas: a 1 cm^3^ volume is composed of 1 cm, 1 mm, and 1 nm cubes [8].

Microinjection moulding has been widely used to mass-produce miniature polymeric devices and/or surface micro/nano structures, such as microneedles for drug delivery [1] and microfluidic devices for diagnostics [2,3]. The base of a microneedle patch is usually a millimetre with a single needle of several hundred micrometres in size, while the tip radius is smaller than 5 µm; such examples also include micro gears, micro-optical connectors, and micro liquid dispensers. These products have an overall weight of the patch of several milligrams or below. The other typical polymeric micro products would be micro/nano scale features on a large substrate, e.g., microfluidic chips. Microfluidic chips usually have tens to hundreds of micron channels for liquid manipulation. Such micro parts and micro/nano scale features are characterised by their very small dimensions and high surface-to-volume ratios. Figure 1 compares the surface area of 1 cm^3^ volume, which is composed of 1 cm, 1 mm, and 1 nm cubes, respectively. The corresponding area increases from 6 cm^2^ to 60,000,000 cm^2^. What does this mean?

**Figure 2:**
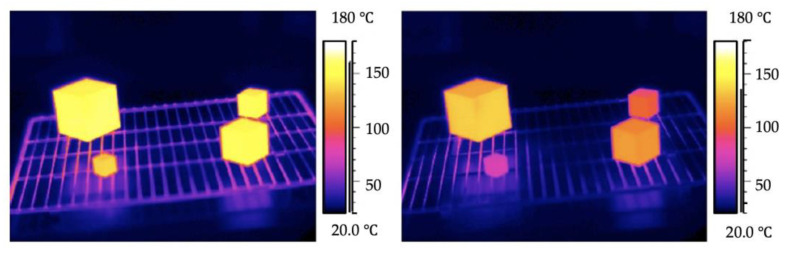
IR images of four aluminium cubes of 20 mm, 30 mm, 40 mm, and 60 mm length near the beginning (**left**) and during the cooling process (**right**) [4].

Let us consider an example, four aluminium cubes of size ranging between 20 mm and 60 mm that were heated in a conventional oven up to ~170 °C [4]. After thermal equilibrium was established, they were cooled down. As shown in Figure 2, it was found that the smallest cubes cooled down the quickest. In general, hot cubes lose their thermal energy via conduction, convection, and radiation.

**Figure 3:**
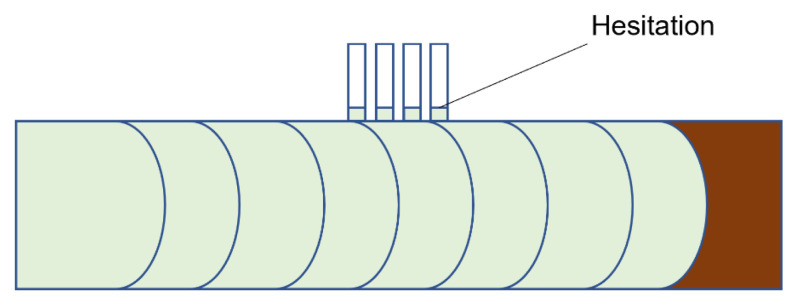
Flow hesitation at the entrance of micro features.

**Figure 4:**
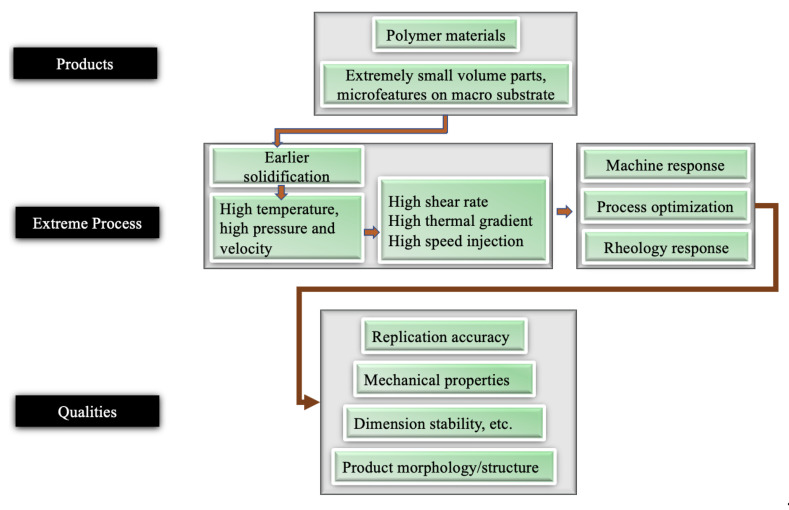
Process-properties relationships overview for microinjection moulding.

When the feature size reduces to the micro/nanometre scale, the surface-to-volume ratio increases by up to 10^4^~10^9^ m^−1^. Once the molten polymer contacts the cold mould, it freezes instantaneously. For example, a 4 µm feature solidifies in 3 micro-seconds [5]. The problem is worse for high aspect ratio features. Additionally, the hesitation effect exacerbates the freezing problem. When a polymer melt is injected into a cavity, it is inclined to flow into the area with less resistance [6]. In a microfluidic device, micro features are designed on the surface. Therefore, flow hesitates (Figure 3) at the entrance of micro features until a much thicker substrate is fully filled. The hesitation duration, that is the “filling time” of the substrate, is usually longer than the critical cooling time of micro features [7]. As a result, polymer melts tend to freeze off at a hesitation point. From a process point of view, high injection velocity and high temperature (including variotherm mould heating) are required to obtain fast filling. Polymer material will be subject to high shear rates and high thermal gradients. Polymer rheology is influenced by possible wall slip and shear heating. Small volume parts require a precise metering injection moulding machine. Process optimizations are challenging because of the short filling time and relatively slow machine responses. Such extreme processes finally influence micro feature replication, product morphology, and its final properties. A schematic of the extreme process and the resulting properties is shown in Figure 4. Knowledge of the evolution of part properties, including surface features under such extreme processing, the resulting microstructure, and performance relationships, are highly desired to secure product quality and process efficiency. Together with micro features and micro parts, the relationships of the machine, process, rheology, micro/nano scale replication, morphology, properties, and applications are detailed in this review.

## 2. Microinjection Moulding

When the development of microinjection moulding started in the late 1980s, no appropriate machine technology was available. Only modified commercial units with hydraulic drive function and a clamping force of usually 25–50t could be used to replicate micro features with high aspect ratios by injection moulding [9]. Using a conventional injection moulding machine to produce micro parts is challenging. From the perspective of processing, decreasing cavity size poses challenges for filling a cavity, especially when cavity dimensions decrease to the micro and nanometre scale. Firstly, due to the decrease in the size of components, the volume of a moulded part decreases to several cubic millimetres, which requires precise metering of a small amount of polymer and fast injection. Conventional hydraulic injection moulding machines have an injection velocity of 200 mm/s. A fully electrical-driven injection moulding machine can achieve more than 600 mm/s injection velocity. A milligram part requires the precise metering capability to accumulate polymer melts less than several milligrams in one shot. The injection screw/plunger has to be scaled down to several millimetres with the precise motion of several micrometres. Some industrial microinjection moulding trials to produce micro parts with conventional injection moulding machines highlighted problems, such as process consistency, long cycle time and waste of material, residence problem (degradation) due to excess material remaining idle in the barrel. Secondly, because of the small stroke of the injection screw/plunger, an injection unit for a microinjection moulding process must respond extremely fast to reach the required injection pressure/velocity. Thirdly, consistency is of paramount importance for microinjection mounding. Micro moulded parts require extremely demanding tolerances, such as for some optical components, up to ±3 µm. A consistent process must ensure proper and repeatable replication of micro/nano features as well as maintaining tolerance. Fourthly, extreme process conditions modify the microstructure and properties of polymers. Additionally, for replicating micro/nano surface features, the combination of all process parameters should make sure that the macro part has no defects, such as short shot, thermal degradation, flash, and at the same time, ensure that micro/nano features can be replicated with high quality and high consistency. Moreover, a specially designed ejection system is required to demould parts smaller than several millimetres in scale, e.g., suction demoulding, air ejection, etc. Progress in variotherm moulding systems has been made in recent years with various heating methods for microinjection moulding applications, such as electrical resistive heating, induction heating, and infrared radiation [10].

### 2.1. Equipment Development

Tremendous efforts have been made in the past 20 years and microinjection moulding machines based on plunger, reciprocating-screw, and multi-stage styles, have been invented [11]. Various precision injection moulding machines have been developed, such as Microsystem50, the newly developed Micropower series 5–15t, Arburg micro injection microinjection module, Desma Formica Plast^@^ one component/two components microinjection moulding machines. Table 1 lists some commercially available microinjection moulding machines and their characteristics. Microinjection moulding machines can be categorized into three types as follows.

#### 2.1.1. Single-Step System

**Figure 5:**
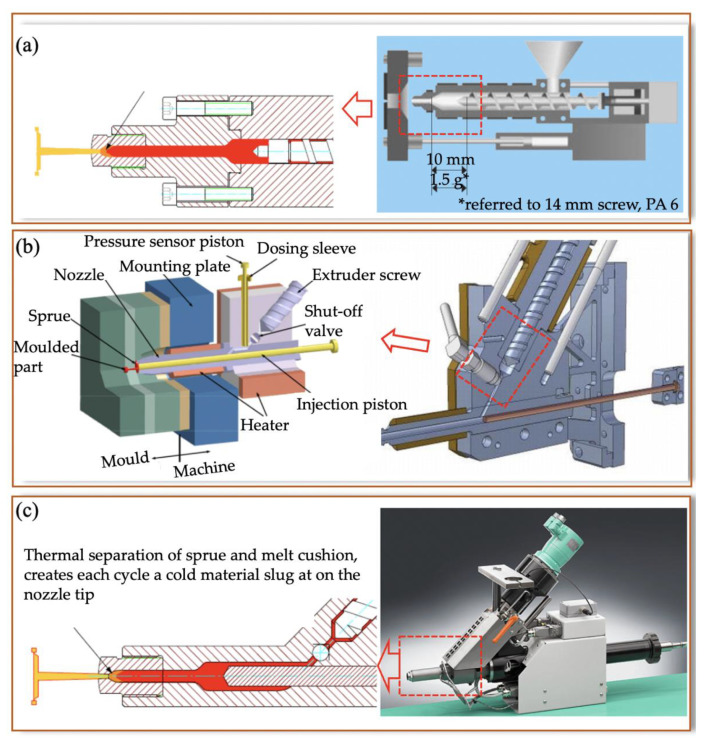
Microinjection moulding system: (**a**) one-step system [15], (**b**) two-step system (Arburg new microinjection module)** [15,16]**, and (**c**) three-step system (Microsystem50) [17,18].

A single-step system is a downscaled technology of a standard injection moulding machine that combines the plasticizing screw and injection piston together. A single-step system reduces the dimensions of the injection unit by reducing the size of the barrel and screw to ensure precise metering and to limit degradation, as illustrated in Figure 5a. High-speed injection and plasticization of standard polymer pellets require a sufficient screw channel depth and sufficient screw strength. As a result, the diameter of an injection screw cannot be smaller than 14 mm in diameter in order to maintain screw life and plasticization efficiency [13,14]. Smaller diameter injection screws can struggle to maintain injection pressure and feed polymer pellets. The typical single-step system commercial microinjection machine is the Fanuc Roboshot s2000i 15B (Yamanashi, Japan). Compared with other microinjection moulding machines, a single-step system has a larger melt cushion and cold material slug, a long flow length, and difficulties in controlling very small shot weights, for example, 1 mg (1 mg shot weight needs ~0.0056 mm stroke on a 14 mm screw).

#### 2.1.2. Two-Step System

A two-step system has a separate plasticizing screw and injection piston. One uses a plunger and hot cylinder, and the other uses a screw and a barrel. If the plunger is only a few millimetres in diameter (3–8 mm), it provides more precise control of the amount of material for the same displacement, compared with a larger screw of a single-step system, as indicated in Figure 5b. It still has a large melt cushion, cold slug, and long flow length.

#### 2.1.3. Three-Step System

A three-step system possesses a split plasticising screw, a metering piston, and an injection piston, as shown in Figure 5c. One uses a plunger and hot cylinder for plasticizing, and the other two use a screw and a barrel for metering and injection. Because of the smaller diameter of the piston for metering and injection, precise control metering and direct injection can be realized without long flow lengths. The benefits of this configuration include a very small melt cushion, no cold material slug, and a very short flow length. The typical three-step microinjection moulding machine is Battenfeld’s Microsystem 50. However, to address cleaning difficulties, Battenfeld’s new generation Micropower 5–15t series modified the design of the metering unit (pressure sensor) and removed the shutoff valve. It also integrated optimal solutions for implementing cleanroom applications.

Control of the final properties of a polymer product starts from raw materials and processing. Knowledge of the dynamic response of the machines and its effect on the injection moulding process is of great importance for milligram parts. Typically, a Battenfeld Microsystem 50 has an injection plunger diameter of 5 mm. This means it can meter very small amounts of the polymer melt (~19.6 mm^3^) when the plunger moves forward 1 mm, equalling 0.023 g of PMMA. A conventional scale-down reciprocating injection moulding machine equipped with a 14 mm diameter injection screw can only meter as small as 154 mm^3^ for 1 mm screw movement, i.e., around 0.183 g PMMA, which corresponds to ~6 standard polymer pellets. As a result, for such a very small part, a moulding machine with a large injection screw has no chance to inject material and switch over to the holding stage due to its small injection stroke. It is also difficult to reach a high set injection velocity. Mould manufacturers usually design the sprue and runner system to be 100 times larger than the micro parts so that industrially conventional machines can control the metering size. Although it can work, it is far from ideal because not only is more resin used, but the production cycle is also extended. Process repeatability is also a problem. Furthermore, machine parameters do not provide as much direct control as the conventional injection moulding process. This means that knowledge from conventional injection moulding is not transferable to microinjection moulding. For example, tuning the process to adjust micro part morphology and enhance the filling of micro/nano features is difficult, since the relatively large sprue and runner hide the effects of machine parameters on the micro part. In contrast to conventional injection moulding, the morphology of microinjection moulded parts has some unique features: high surface-to-core ratio, highly oriented, and irregular morphological features. These features result from high shear rates and high thermal gradients. Effectively, control and optimisation are based on process characterisation, which is the key to controlling the final properties of a product. Although this has been recognised, relatively little effort has been made to create process-specific and material-specific knowledge.

### 2.2. Process-Rheology and Crystallisation and Morphology of Micro Products

As discussed in the introduction section, for injection moulding of micro features and micro parts, the extreme process is involved as high shear rate and high thermal gradient, which will influence the morphology and properties of micro parts and micro/nano features. Recent research mostly focuses on the study of crystallisation and morphology-determined mechanical properties of micro products. Thus, the discussion here covers a part of rheology and more on crystallisation, morphology, and mechanical properties.

#### 2.2.1. Rheology of Polymer Material at Micro Scale

**Figure 6:**
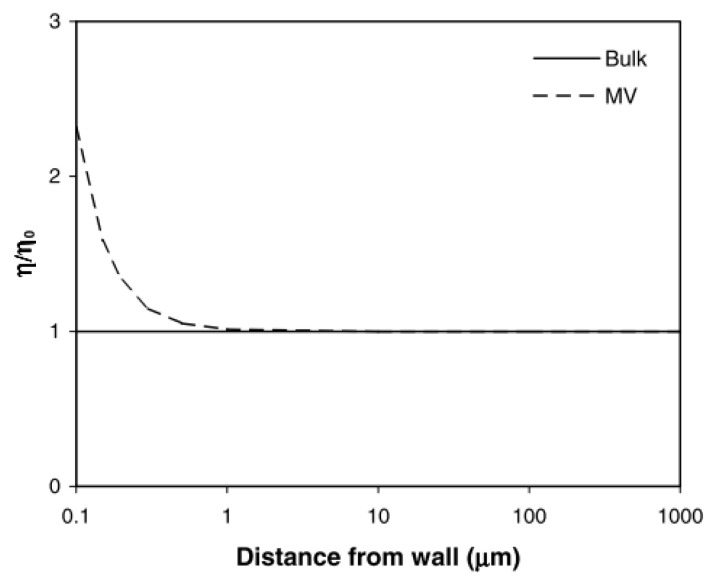
Micro scale viscosity (MV) was predicted by the Eringen-Okada equation [28].

Rheology means flow and deformation [19]. Understanding polymer melt rheology at the micro/nano scale is important for quality control, process design, and simulation of micro/nano features [20]. It was evidenced that liquids such as water, silicon oil, alcohol, and polymer solutions flowing in microchannels with characteristic dimensions of tens of micrometres had a viscosity of 50–80% close to the channel, where the viscosity was higher than that of the bulk fluid [21]. The higher viscosity increase was attributed to collective molecular motion effects or to the immobility of the layer of molecules in contact with the solid surface [22]. Generally, techniques such as rotational, capillary, or slit flows are used to obtain accurate measurements at a series of set strain rates and temperatures [23]. Some works have been done by using slit/capillary dies embedded into either a nozzle or mould to test the material rheology with an injection moulding machine or an extruder [24,25,26,27]. This method can provide rheological data under the same thermo-mechanical history that is experienced by the testing materials during real process conditions, such as plasticisation and viscous heating. Efforts have been made in the last ten years to understand polymer rheology at the micro scale. Yao and Kim [28] initially simulated the filling of a microchannel to include the micro-rheology and power-law slip model. A micro scale viscosity model was introduced by Eringen and Okada [29],

(1) $$\eta =\eta_{b}[1+\xi {\left(g\;/D\right)}^{2}]$$

where *η_b_* is the bulk viscosity disregarding the inner structure of the fluid, *ξ* is the non-dimensional constant, *g* is the gyration radius of fluid molecules and *D* is an external dimension. Figure 6 compared micro scale viscosity and bulk viscosity corresponding to different dimensional scales. Viscosity significantly increased when the channel dimension was reduced to less than 1 µm.

Additionally, viscous fluids adhere to attain the velocity of the boundary during flow. However, a relative velocity exists at the contact line between the fluid and solid boundary during flow: this is the so-called “wall slip” [30]. Slips of polymer melts are explained by flow-induced chain detachment/desorption and chain disentanglement. The various experimental methods of determining wall slip velocity can be found in an excellent review [31]. In microinjection moulding, polymer melts are subject to very high shear stresses, which can easily exceed the critical shear stress. Since a cavity is reduced to the micro scale, the effect of wall slip would be more significant than the conventional injection moulding.

**Figure 7:**
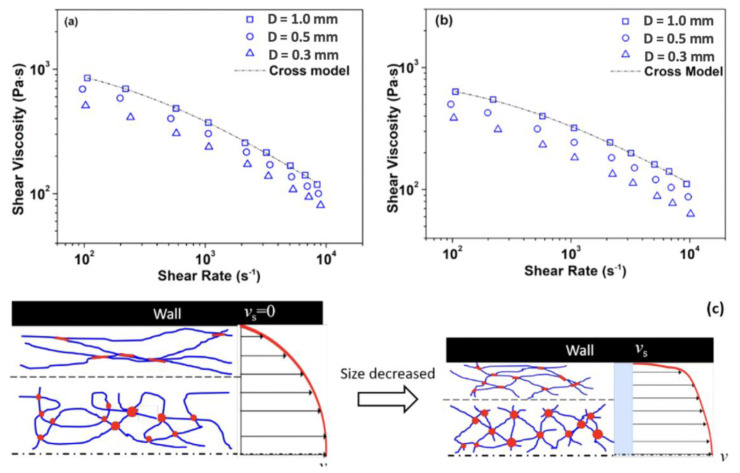
Variation of shear viscosity for different micro channels of (**a**) neat PC, (**b**) PET50, and (**c**) schematic diagram of “shear−thinning” under micro scale [33].

The rheological behaviour of polymer melts under extreme process conditions in microinjection moulding has attracted considerable attention. Figure 7a,b demonstrates the influence of shear rate on the viscosity of polymeric dies. When the size of the flow channel was in the micro scale region, a scale-effect determined rheological behaviour always exists. With reducing capillary diameter, the viscosity decreased with the constant shear rate. This was due to the wall slip effect-induced disentanglement/entanglement mechanism, which was shown in Figure 7c. Trotta et al. [32] focused on the thermo-rheological behaviour of polymer melts in micro channels where the mould temperature was lower than melt temperature, indicating that the heat transfer analysis of conventional injection moulding was not applicable to the microinjection moulding of a very thin part with a very large surface-to-volume ratio. Although these studies give some insight into polymer rheology behaviour at the micrometre scale, they either focused on viscosity measurement under assumed isothermal conditions or they did not separate the effects of plasticisation, wall slip, and non-isothermal conditions when analysing rheological behaviour.

#### 2.2.2. Crystallisation and Morphology Development

**Figure 8:**
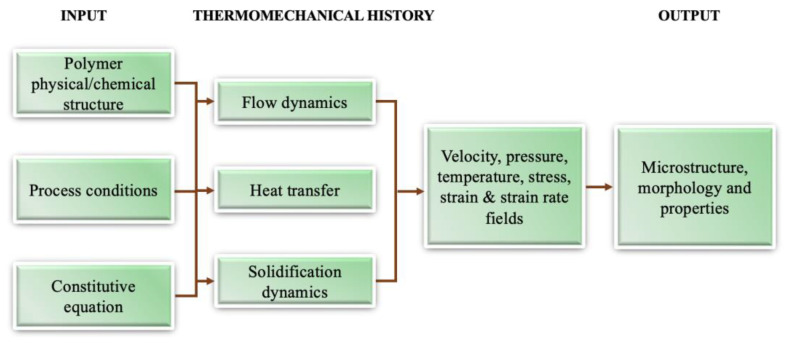
Components of a polymer processing system.

Morphology is the study of form and structure. Polymer morphology is the study of order within macromolecular solids. The thermomechanical history experienced by polymer materials in their processing imparts to its microstructure (crystallinity, morphology, orientation, and residual stress, etc.), as shown in Figure 8. It is evident that the three transport phenomena (flow, heat transfer, and crystallisation kinetics) are involved in structure formation during processing [34]. Flow causes macroscopic heat and momentum transport. Meanwhile, it also influences crystallisation kinetics by controlling stress, strain, and strain rates. This microstructure will ultimately determine the product properties (mechanical, optical, and barrier, etc.). As a result, characterisation of multiscale microstructure and prediction of microstructure at micro, nano, and/or molecular scales are important aspects of polymer processing research. Microinjection moulding refers to miniaturized parts or micro/nano scale features. In this process, the material will experience a very high shear rate, injection pressure, and thermal gradients. These extreme process conditions will create a special morphology. This section will review prior work on characterising morphology evolution for both micro parts and micro features and final properties.

(1) Crystallisation and crystal structures

Crystallisation is a phenomenon that takes place when a polymer, having the ability to be ordered (chemical and structural regularity), is cooled below the equilibrium melting point [35]. Crystallisation under quiescent conditions is a phase transformation process, which is caused by lowering the temperature or by changing the pressure from a dilute polymer solution or a molten polymer. Under flow conditions, polymer chains are extended by shear or extensional strain, which could increase opportunities for crystal formation by providing more nucleation precursors [36,37].

**Figure 9:**
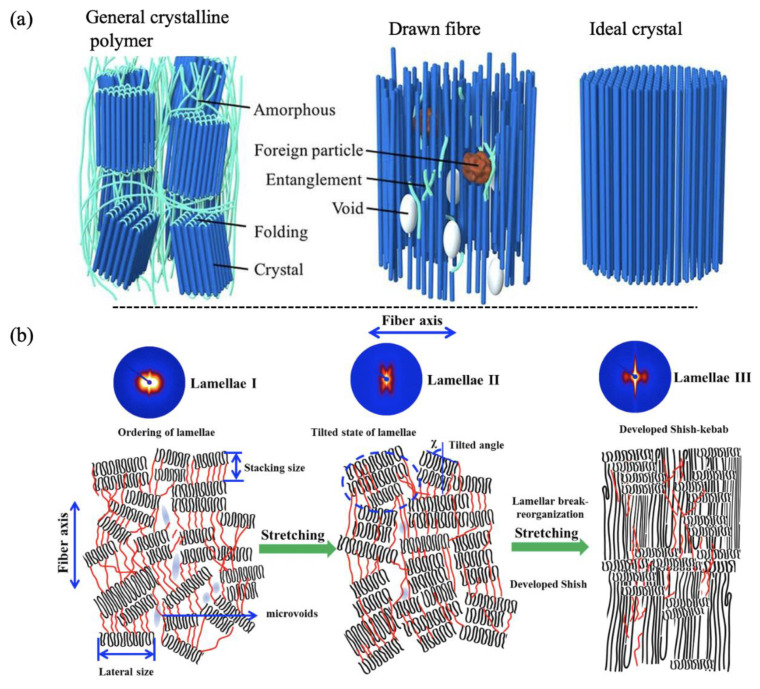
Schematic representation of the molecular structures: (**a**) a general crystalline polymer and a drawn fiber, and an ideal polymer crystal [43] and (**b**) lamella break-reorganisation process and development of shish-kebab [48].

Depending on the polymer molecule conformation, there are two dominant morphologies in semicrystalline polymers: spherulites and fibril-like shish-kebabs [38,39,40]. Random coiled polymer chains will form thin platelets whose large upper and lower surfaces consist of an array of molecular folds, which are called “chain-folded lamellae” [41,42]. Figure 9a shows the molecular structures of a general crystalline polymer, a drawn fiber and an ideal polymer crystal. The spherulite is composed of stacks of ribbon-like crystals, which grow outward radially from a single (heterogeneous) nucleus from a group of lamella similar to that in heritage, or a quadrate (a crosshatched lamellar array) [43]. Ribbon crystals have a thickness in the order of 10 nm, in which polymer chains are arranged approximately parallel to the thin dimensions of the ribbon. The inter ribbon region is composed of amorphous tie chains, dangling chain ends, totally unincorporated chains, and loose loops. Single lamella crystals consist of a crystal lattice, which is composed of an arrangement of an individual atom. Generally, bonding along the chain is covalent, while bonding perpendicular to the chain is van der Waals force or hydrogen bonds, which are much weaker. The size of spherulites ranges from 50–500 µm, which is the largest domain with a specific order. This size is much larger than the wavelength of visible light, making semi-crystalline materials translucent and opaque [44].

Shish was found more stable than spherulites, with a melting temperature of 15–20 °C higher [45]. Therefore, it was considered that shish was formed by the crystallisation of fully stretched/extended chains. The kebabs were believed to be folded chain lamellar structures [46]. Kebabs were normally grown from shish and the chain alignment in the kebabs was believed to be parallel to the shish. The degree of stress during crystallisation influenced the nature of folded chain lamellae which grew from the fibril nuclei and comprised the bulk of the crystallised specimen [29]. As indicated in Figure 9b, high stress promoted the growth of lamellae of planar conformation, while low stress resulted in a twisted nature [47].

(2) Crystallisation kinetics under quiescent conditions

It is well known that the crystallisation process is composed of three stages: nucleation, growth, and perfection [49]. R. Iervolino [50] gave a brief description of Hoffman–Lauritzen’s theory for explaining the crystallisation of flexible polymers. During the crystallisation process, the relaxation of metastable undercooled melt transfers towards the equilibrium state. This needs to overcome a free-energy barrier, which depends on the degree of undercooling. Interfaces have to be introduced into the metastable melts to create a new phase. If the resulting cluster, originated by the reactions of association and dissociation of chain segments, has a size smaller than a critical one, it is unstable. For example, its probability of decrease is higher than its probability of growth. On the other hand, if a critical size is exceeded, the nucleus is more likely to grow than to decrease. This kind of nucleus, called active, can continuously grow toward a stable crystalline phase. If nucleation is initiated from a single phase, it is called homogeneous nucleation. Homogeneous nucleation generates primary nuclei without the help of any substrate or external nucleating particles, such as nucleation agents. If the process is initiated from multiple phases, heterogeneous nucleation occurs. In this case, the nuclei are formed on the surface of foreign bodies or crystals of the same material already present in the undercooled liquid. In practice, homogeneous nucleation is an unusual and unlikely event. Heterogeneous nucleation takes place in most cases. Obviously, a nucleation process under quiescent conditions is highly influenced by temperature, pressure, and the presence of nucleation agents.

However, the Hoffman–Lauritzen theory is unable to explain the self-nucleation effect, which was discovered by Bulndell, Keller, and Kovacs [30]. H. Janeschitz–Kriegl et al. [51,52] studied the relaxation of thread-like precursors, and found that nuclei were quite stable for a relatively long time depending on the temperature when the temperature exceeded the melting temperature of spherulites. This provides a way of explaining self-nucleation.

The Avrami equation provides an easy way for obtaining the bulk crystallisation kinetics information [53,54]:

(2) $$x\left(t\right)=1-e^{-kt^{n}}$$

where *x*(*t*) is the relative crystallinity at time *t* with *t* = 0 corresponding to the end of the induction period. The Avrami exponent, *n*, ranges from 1 to 4 and depends on the type of nucleation, growth geometry (rod, disk, sphere), and growth control mechanism. For example, the Avrami exponent for spherulitic growth from sporadic nuclei is around 4 [55]. If the growth is activated from instantaneous nuclei, the Avrami exponent is lowered by 1.0 for all cases. The crystallisation rate, *k*, depends on the product of the rates of two processes: nucleation and crystal growth.

(3) Flow-induced crystallisation

**Figure 10:**
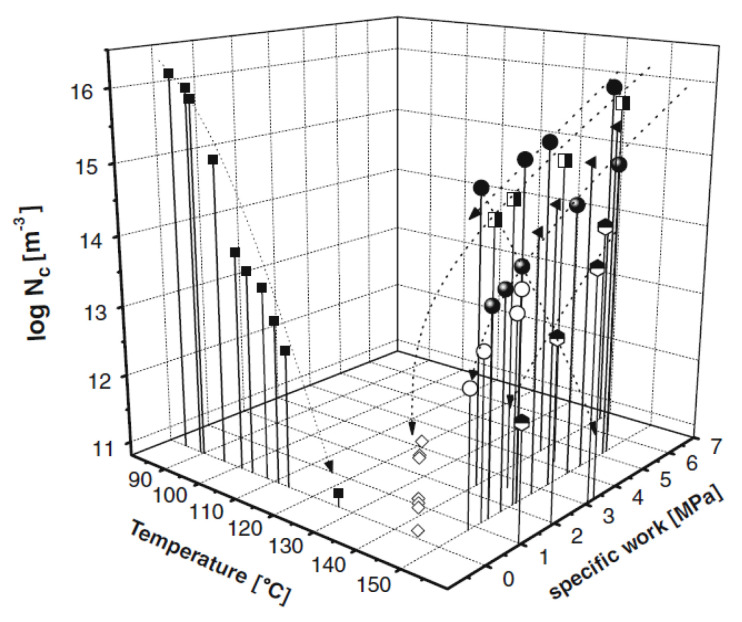
A three-dimensional plot of the (logarithm) of the number density of nuclei (m^−3^) against temperatures after fast quenching (left horizontal axis) and against applied specific mechanical works (MPa; right horizontal axis) for an industrial-grade of PP [58].

Crystallisation of polymer melts under flow conditions has been extensively studied, since flow and pressurisation are involved in almost all polymer processing methods. Semicrystalline polymers represent two-thirds of all synthetic polymers. The morphology and properties of their products depend on the manner of the polymer crystallisation from a flowing melt [56,57]. Figure 10 summarises the effect of flow on crystallisation. Specific mechanical work is the amount of shear that has been applied to a polymer melt, which will be discussed later in this section. It can be seen that the number of nuclei could be enhanced by many orders of magnitude if shear or extensional flows were applied for short periods to polymer melts [58]. Shear effects is equivalent to temperature for the nucleation process.

**Figure 11:**
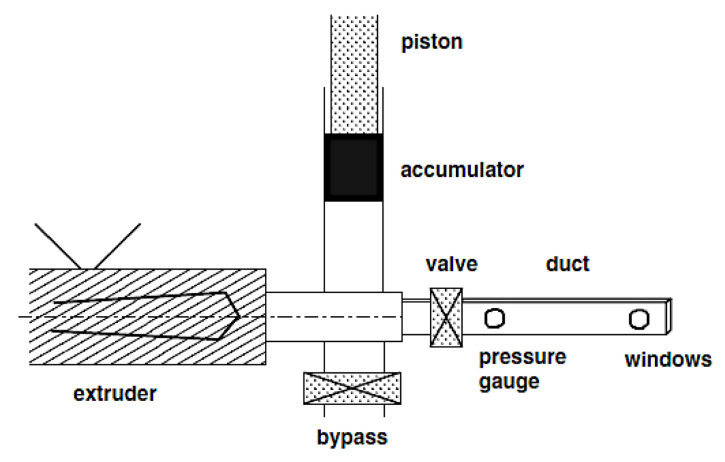
Schematic presentation of the arrangement for the short-term shearing experiment [60].

In the last five decades, many efforts have been made to understand flow-induced crystallisation experimentally. Janeschitz–Kriegl et al. [34,59,60] introduced a novel “short-term shear” protocol with designed instruments to separate the effect of shear on primary nucleation from crystal growth, and the principle was then used by many groups to study shear-induced crystallisation [34,61,62,63,64]. In the “short-term shear” protocol, shear was introduced by a piston with weight on the lever [60], a pneumatic actuator [65], or a high torque stepper motor [64], etc., all of which were able to control shear action and shear time. As shown in Figure 11, polymer melts were firstly filled into the cavity over their equivalent melting temperature to erase their thermo-mechanical history and then were cooled to the target crystalline temperature after molecule relaxation. Shear was then applied for a period afterward, following quiescent crystallisation. This process had a similarity to the injection moulding process, but in which thermomechanical history was not erased, and hold pressure had also been applied to compensate for material shrinkage.

**Figure 12:**
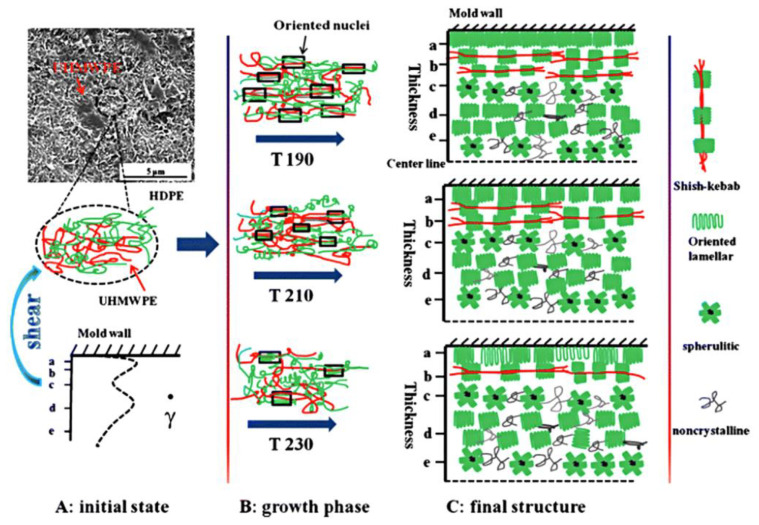
Schematics of the formation of skin-core structure [66].

The morphology of a part made by such protocols showed several distinct layers of “skin-core“ morphology of a typical extruded part and injection moulded part, as shown in Figure 12. The skin layer was composed of oriented structures, followed by an oriented “fine-grained” layer and an isotropic spherulite core. Such morphology was tightly related to the deformation history experienced by the polymer molecules under a combination of shear rate and shear time. Firstly, the polymer melt was assumed to have a large reservoir of dormant nuclei [34]. These nuclei can be activated by quenching and specific work (defined as energy density from external shear), indicated by a significant increase in the number of nuclei [58]. Initially, nuclei could be point-like, depending on local stress, although this point is still controversial. Thread-like nuclei (pre-stages of so-called “shishs”) can be grown if the melt is sheared heavily. However, thread-like nuclei are not stable and tend to relax, depending on temperature and molecular chain length. The density of nuclei under flow conditions finally determines the crystal structure. Point-like nuclei under quiescent conditions are inclined to form spherulites, and thread-like nuclei can either form highly oriented structures or spherulite structures if there is enough space.

**Figure 13:**
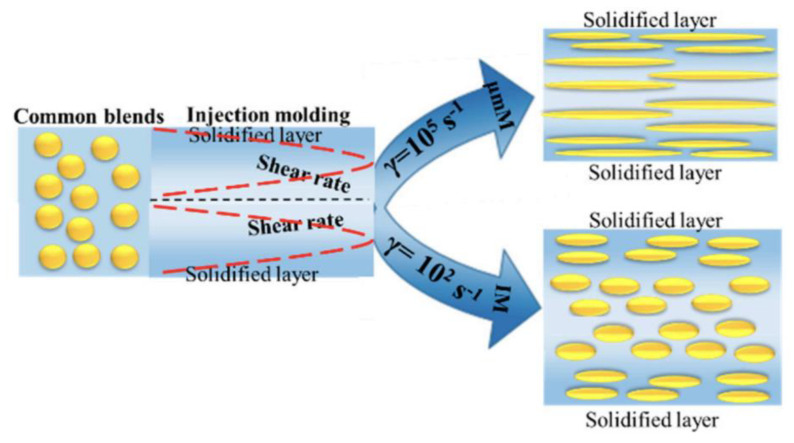
Schematic of the mechanism of morphology evolution under shear effect during microinjection moulding [69].

Recent research into shear-induced crystallisation confirmed the boundary flow conditions for the onset of oriented morphology [39,64]. An oriented structure can be formed once polymer melts crossed a threshold value, like shear stress, shear rate, strain, specific work, and their combinations [39,58,60,61,64,67,68]. However, it is still unknown how best to define such thresholds or how they depend on the molecular attributes of a polymer [40]. The mechanism of shish formation is still controversial. Kornfeld’s group put forwarded the phenomenological explanation of the formation of oriented structure based on shear stress [40,68], as shown in Figure 13. For a given material, there is evidence of a threshold stress σpt above which shear flow can induce the formation of “point-like nuclei” and below which shearing has negligible effect. Another critical shear stress σskin > σpt is associated with the formation of “thread-like nuclei”. The thread-like precursors will provide a surface for the growth of radially oriented lamellae, so-called “kababs”. Except for the critical shear stress, specific work was used as a threshold for the formation of oriented structures by many researchers [39,62,64]. Mykhaylyk et al. [64] gave a possible physical explanation of specific work: upon straining for a time dt, the melt experiences the stress σ=ηγ and the strain dγ=γdt; and the minimum total work ω=∫σdγ, in which the stress extended the molecules and led to a higher probability of stretched chain segments.The long chains were proven to be playing an important role. It was reported that long chains accelerated the formation of shish. Two-time scales, long-chain rouse time t_R_ and short-chain disengagement time t_D_, are important to largely determine whether the long-chain will undergo chain stretching [40,62]. Flow can orient polymer chains at the shear rate $\gamma >1/t_{D}$ and the stretching of polymer chains at the highest shear rates $\gamma >1/t_{D}>1/t_{R}$. While the first condition could be necessary to form an oriented structure, the second condition ensures strong stretching of polymer chains [64]. An important saturation of “point-like nuclei” was detected by light scattering [68] and rheological measurement [62], indicating that the number of nuclei was independent of shear time. It probably led to the change of growth of an oriented structure from highly sensitive to flow to a mechanism independent of flow. However, it has not yet been possible so far to develop a method for determining the influence of flow on the growth speed of the crystalline domain [34].

The structure of spherulites and shish-kebabs and their relative proportions in a polymer’s morphology are some of the main factors that are responsible for the mechanical properties of commercial products. The formed structures distinctly influence the properties of moulded parts. The resulting microstructures are distinct from those generated under quiescent conditions, bringing about the formation of anisotropic morphology, named ‘skin–core’, within the thickness of the parts [64]. This skin-core morphology is in fact composed of four different layers within the thickness. For micro parts, the thickness can be reduced to a few hundredths of a micron. The moulding step resembles injection moulding. The main distinguishments for microinjection moulding lie in the higher temperature for polymer melt and mould, and the higher speed and pressure during moulding. A shear thinning effect would theoretically reduce the polymer viscosity, causing a higher filling length inside the micro cavity [69]. However, the rheological behaviour of flow in micro cavities differs from conventional rheology laws. In the microinjection moulding process, many factors such as the high shear stress, mould temperature, cooling rate, etc., can significantly affect the nucleation and growth rates of crystals. The modulation of process parameters results in forming various microstructures and crystalline structures, thereby influencing the mechanical properties of the micro parts. Overall, microinjection moulding is being utilised as a platform to investigate the crystallinity and evolution of phase structures, such as super molecular structures (shish-kebab, transcrystalline, or β-cylindrite), in a complicated flow field.

(4) Morphology development and product properties in microinjection moulding

**Figure 14:**
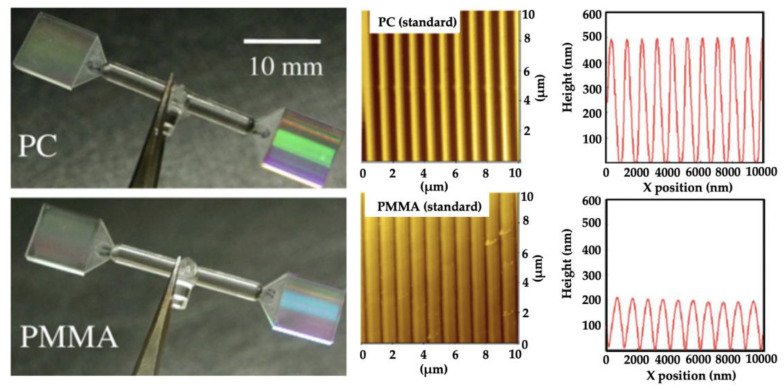
Microinjection moulded parts (0.3 mm) with nano-grove features [79].

**Figure 15:**
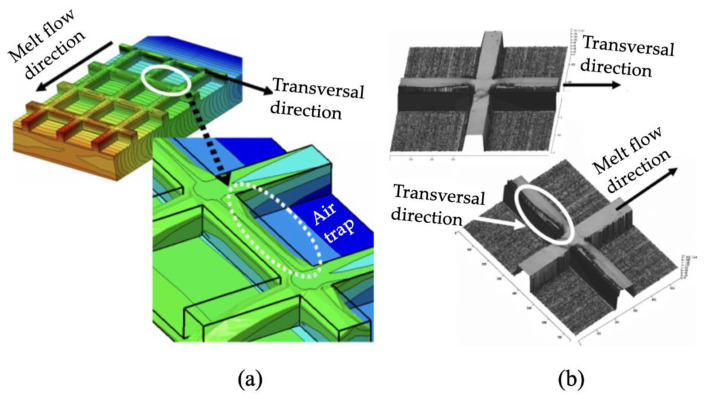
Comparison of air traps calculated by numerical simulation (**a**) and by AFM measurement (**b**) [80].

The skin-core morphology of microinjection moulded parts has been reported by several authors in the past 15 years [70,71,72,73,74,75]. The following section reviews the progress of morphology studies of microinjection moulding, and identifies the morphology differences between microinjection moulding and conventional injection moulding. The earliest study known to this author into the morphology of microinjection moulded parts was started at the University of Bradford [76,77]. They initiated process measurements, process repeatability studies, and morphology and properties study for polymeric microinjection moulded products. Ito et al. [78] studied the morphology of PP considering the effect of mould temperature and mould thickness, using WAXD, DSC, polarized light microscopy (PLM), and birefringence measurement. The skin-core structure was detected by PLM, although no spherulite structure at the core region was observed with a thin cavity at a low mould temperature. The molecular orientation increased with the decrease of cavity thickness. They also explored the combination of thin wall micro parts with microfeatures [79]. Micro squared parts (9 × 9 mm) with 400 × 100 µm grids and line features sized from 50 µm to 200 µm were moulded using PC and PMMA. The microfeatures were fabricated by a stainless ferrochrome alloy using diamond turning and micro-milling. Nanoscale lines were fabricated by electroplating a Si master with features patterned by electron beam lithography. Depending on the replication of nano features, interference colours were different for PMMA and PC, even though the same master mould was used, as shown in Figure 14. Additionally, the replication ratio varied from the gate to the flow end, which was attributed to cavity pressure distribution. For their observation, the thicknesses of these morphological layers were affected by moulding conditions, and the surface patterns were influenced by the skin layer. However, the relationship between feature replication and its morphology distribution is still not clearly understood. Recently, they used 3D numerical simulation to explore the mechanism of surface pattern replication [80]. They found air entrapment in the filling stage had a strong relationship to replication shape and replication rate, as shown in Figure 15.

For mechanical properties, Haberstroh et al. [81] designed a micro tensile tester to characterise the effect of dimensions on the mechanical properties of a micro tensile specimen. They found that no uniform correlation between the miniaturisation of the tensile bar geometry and the changes of the mechanical properties. They believed that differences in stiffness and strength resulted from internal properties such as structure fineness, degree of crystallinity, orientation, and internal stress. They proposed that micro parts should not be dimensioned using mechanical properties determined at macro geometries. However, this has not yet been confirmed.

**Figure 16:**
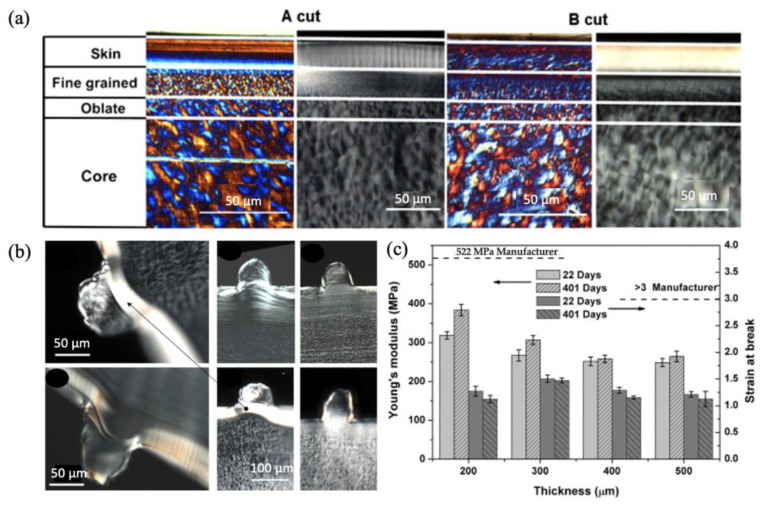
(**a**) Morphology distribution at the cross-section of miniaturised parts, (**b**) morphology of 100 μm features, and (**c**) the effect of cavity thickness on Young’s modulus [82].

Zhang et al. [82] investigated the effect of the gate design and cavity thickness on filling, morphology, and mechanical properties of Poly(ether-block-amide) miniaturised parts. Micro parts presented the typical “skin-core” morphology, no matter where they were cut along the transverse or flow direction, as shown by polarised light microscopy in Figure 16a. Figure 16b demonstrates the surface morphology of microinjection moulded Pebax and HDPE features parts with 100 μm feature size. HDPE features at the gate and part end presented an analogous microstructure to the skin layer, implying that during the feature filling stage, the shear rate was sufficient to generate the oriented structures. It noted that severe feature deformation occurred near the gate of the part. This may be due to the incomplete feature filling in the cavity or the skin layer compressed into the cavity under the melting force. However, the feature deformation at the end of the part was ignorable owing to the low melt pressure. Figure 16c indicates that the increasing cavity thickness enables the reduction of Young’s modulus, companying the decrease of skin ratio and orientation factor. As the cavity thickness increased from 200 μm to 400 μm, the strain of the part increased first and then reduced. In addition, elongation is considered to be associated with skin ratio and molecular orientation. Importantly, it notes that a critical dimension exists, over which microstructure variation has a more significant effect than the skin ratio or molecular orientation.

**Figure 17:**
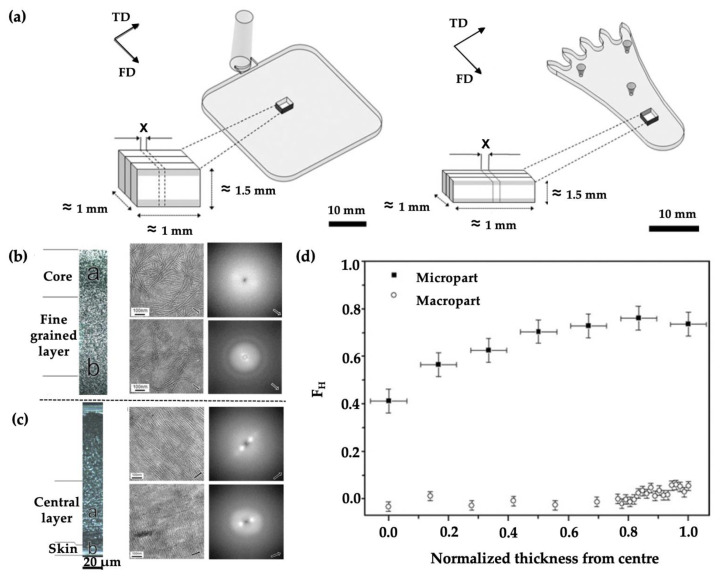
(**a**) The geometry of the macro part (1.5 mm thick) and micro part (150 µm thick), morphology examined by PLM, TEM, and WAXD for (**b**) macro part and (**c**) micro part, and (**d**) distribution of molecular orientation of the macro part and micro part [74].

Giboz et al. [74] compared the morphology of a micro part (thickness: 150 µm) and a macro part (thickness: 1.5 mm), which was made with HDPE by the microinjection moulding process, as shown in Figure 17a. The macro part presented a skin-care morphology, having four distinct morphology layers: skin, shear, fine-grained, and core layers; no significant molecular orientation was found for all the morphological layers, as indicated in Figure 17b. The micro part exhibited core-free morphology and only its central region had an oriented structure, as indicated by WAXD in Figure 17c, where lamellae were oriented perpendicular to the flow direction. They also indicated that lamellae of the micro part were thinner than the macro part. Herman’s orientation factor, FH, was used to define the molecular orientation of crystals, as indicated in Figure 17d. The micro part was much more oriented than the macro part along the thickness direction. The maximum orientation factor of the micro part was located at the transition layer, which indicated that additional flow strength occurred in this region. The minimum orientation was found at the centre of the macro part, which had spherulitic morphology, while for the micro part, the value FH = 0.4 indicated that orientation still existed in the central layer.

Jungmeier et al. [83] designed a series of tensile specimens with various surface-to-volume (*s*/*v*) ratios to characterise the effect of the cooling rate on morphology and mechanical properties. Non-nucleated PA66 (Ultramid A3K) and non-nucleated polyoxymethylene (POM Hostaform C9021) were used as moulding materials. Micro tensile specimens were scaled from a standard tensile bar with a scale factor of 1:2, 1:4, 1:8, to 1:16. The crystalline structures of PA66 in the core became finer, with an increase in the surface to volume ratio (*s*/*v*) which was caused by the increase of thermal nucleation due to an increase in cooling rate. The crystallinity of PA66 reduced from 1:2 to 1:8, which was explained by different cooling speeds. A faster cooling was accompanied by a nucleating effect, but crystalline growth was restrained, resulting in a lower degree of crystallinity. For POM, the skin ratio significantly increased with the increase of the *s*/*v* ratio, but crystallinity remained consistent, which was explained by POM having a higher rate and ability to crystallise.

**Figure 18:**
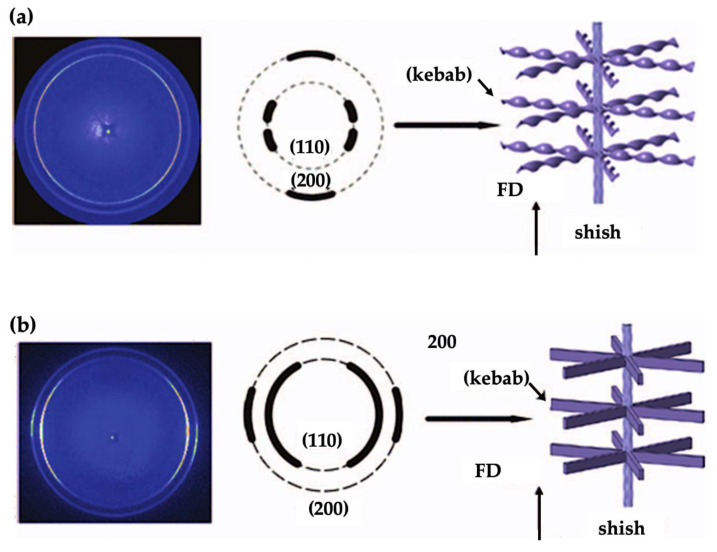
2D-WAXD patterns for a macro part (**a**) and micro part (**b**) [71,84].

**Figure 19:**
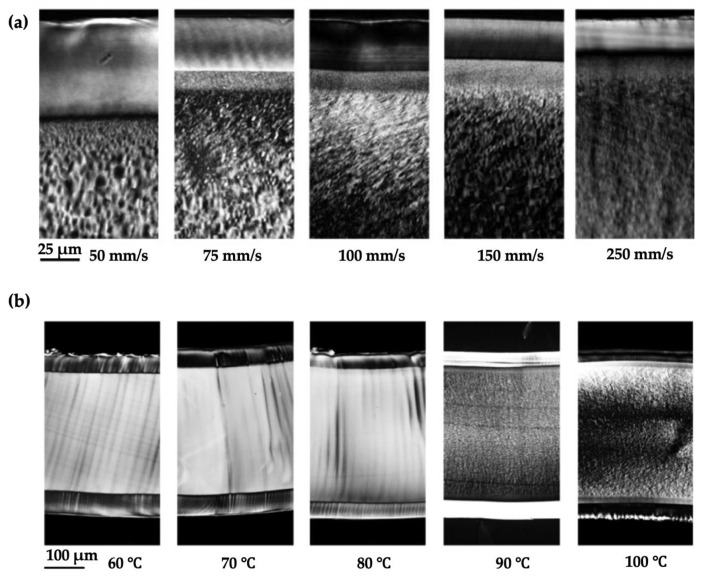
Morphology under various (**a**) injection velocities and (**b**) mould temperatures [85].

Liu and Guo et al. [71,84] also compared the morphology of the micro part (200 µm thick) and macro parts (2 mm thick) made by microinjection moulding using HDPE and PP. A common “skin-core” structure was found for both. The micro part presented a relatively large fraction shear layer, which was attributed to the combination of higher shear rates and faster cooling speeds. The shear layer contained highly oriented shish-kebab structures, and the core layer was composed of spherulite crystals for both HDPE and PP. Their 2D-WAXD characterisation indicated that a twisted shish-kebab structure was formed for the macro part for HDPE in the shear layer, while an untwisted shish-kebab structure occupied the shear zone of the micro part, as shown in Figure 18. The formation of such morphology was attributed to the degree of lamella stretch, which was affected by the number of shish and configuration of kebabs. They also claimed that the volume reduction of the micro part compared to the macro part-induced high shear rates and high cooling rates, which may promote the formation of this special untwisted oriented morphology. It was explained that the micro parts had a high degree of nucleation density, which was due to the high surface-to-volume ratio and flow-induced crystallization. Zhang et al. [85] investigated the effect of flow-induced crystallization on the morphology evolution of Poly(ether-block-amide) using the microinjection moulding process. The morphology distributions are shown in Figure 19. A spherulite-free core structure was generated under the condition of mould temperature within a range of 80–90 °C. The skin layer increased with the increase of injection velocity, and reduced as the mould temperature was raised.

### 2.3. Tooling

Tooling is critical for microinjection moulding. Let us take the microfluidic device as an example. Microfluidic devices are composed of a large substrate, which is the size of a typical credit card (85.60 × 53.98 mm) or a microscope slide (75 × 25 mm) and contains a large fluid inlet and outlet chambers of millimetre-scale, as well as microchannels of tens to hundreds of micrometres in which various fluids are transported and manipulated. Specific features (e.g., micropillar arrays, submicron features) are integrated into the channels for cell separation or for modifying surface properties, etc. The general tolerance is an order of magnitude smaller than the dimensions of the various features, ranging from micron to submicron levels. For mass production of a commercial microfluidic chip, tooling technologies combining multi-scale features are required at a reasonable cost, because the quality and performance of the replicated microchip are mainly dependent on the quality of the corresponding micro structured mould. Hybrid tooling, having multi-scale features, can be realized by several manufacturing processes. Table 2 compares a variety of techniques for manufacturing micro moulds or inserts.

**Figure 20:**
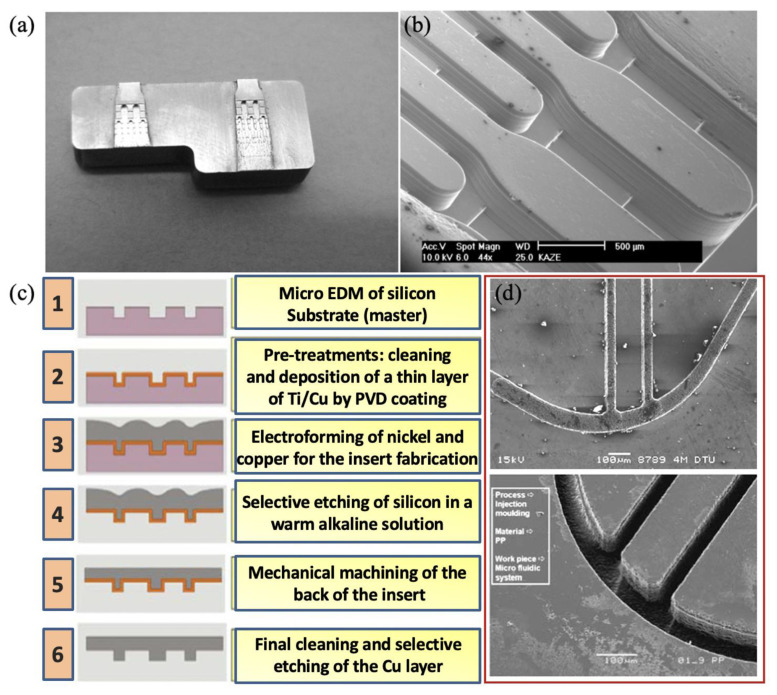
(**a**) Details of microchannel network of electroplated nickel mould insert and (**b**) microinjection moulded part for agglutination assays [93], (**c**) process chain, and (**d**) nickel mould insert and microchannels on microinjection moulded part [95].

**Figure 21:**
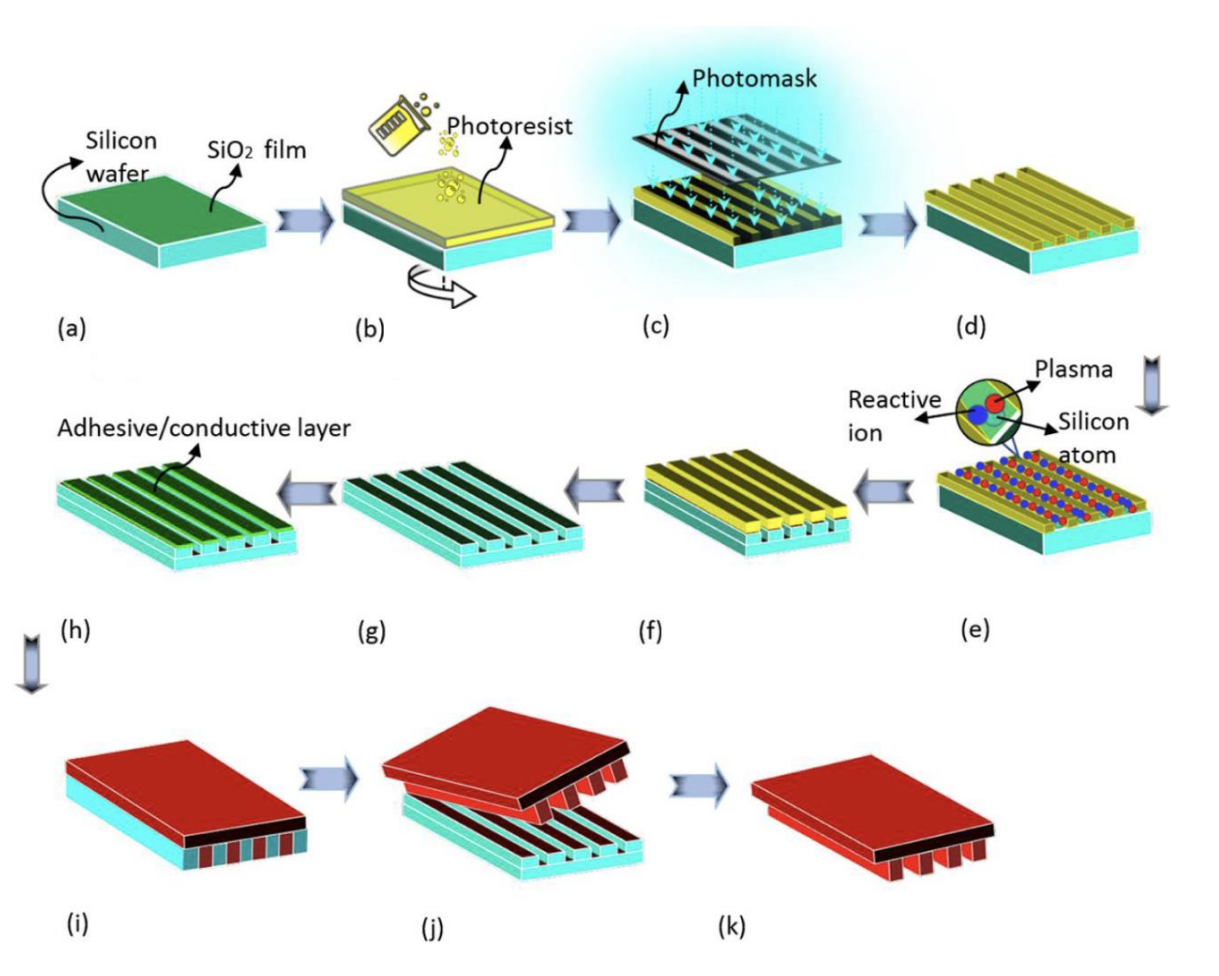
Processing steps required for preparing nickel mould insert: (**a**) silicon wafer preparation, (**b**) spin coating of photoresist, (**c**) exposure, (**d**) development, (**e**) silicon etching, (**f**) etched silicon with the photoresist, (**g**) patterned silicon, (**h**) metallization, (**i**) electroforming, (**j**) demoulding, and (**k**) electroformed nickel mould [96].

For mass production, especially injection moulding, stainless steel can resist wear or other forms of surface or structural degradation over several thousands of moulding cycles, and is a good tool candidate from the perspective of wear and tool life [87,88,89]. Direct machining using micro-manufacturing methods, such as micro milling and micro electro-discharge machining, takes a very long time to machine macro features. Combining conventional machining with micromachining could reduce the total machining time, but it would generate more roughness and burrs. A similar phenomenon can also be evidenced by Nguyen et al. [90,91,92] when they fabricated aluminium shim using the micro milling technique. Additionally, moving a workpiece from one machining process to another could lead to a loss of precision. Figure 20a shows a hybrid mould insert that has large reservoirs and channels of which the smallest measure 40 × 40 µm. The outer geometry and large reservoir are manufactured by precision machining. The small channels were generated by lithography and electroplating [93]. The corresponding replicated feature on plastic is shown in Figure 20b. Some innovation process chains were used to combine micromachining with electroforming for fabricating the mould insert [86], as shown in Figure 20c. The fabricated nickel mould insert and microinjection moulded part show in Figure 20d. This process chain overcame the problems of positioning errors, but it did not allow features smaller than 10 µm to be machined, although it did have a high-quality surface finish. It is also a challenge to machine electrodes as small as 10 µm. Semiconductor-based processes are commonly used in laboratory environments to prototype microfluidic devices. The schematic for semiconductor fabrication routines for a mould insert is shown in Figure 21. The process starts with a lithography step using a mask that transfers the desired pattern into a photoresist. After developing the resist, the underlying conductive layer is exposed, which serves as a plating base for another metal that fills the voids left by the resist that was previously removed by exposure to the patterning radiation. The deposited metal forms the desired microstructures. The unexposed resist is then removed, forming the finished mould master [94]. Nickel and its alloys are common materials for mould inserts. However, access to semiconductor facilities is expensive and time-consuming. Manufacturing of mould inserts for a microfluidic device should be undertaken with consideration of the functional requirements of the device and the cost.

Although stainless steel is the predominant mould material, it cannot be patterned down to the submicron scale, due to the intrinsic grain size of the material. Grain voids, grain boundaries, and crystallographic disorientation could interfere with surface patterns, leading to cracks and plastic deformation. Additionally, current micromachining technology is capable of accurately manufacturing patterns only as small as ~20 µm and leaves machining marks and burrs, resulting in a poor surface finish. Combining two or more direct processes can cause alignment problems when changing from one process to the other, which leads to a loss of accuracy. Electroformed nickel features based on metallic substrates are of particular interest because they offer dimensional precision with a high-level surface finish [97,98]. However, it is difficult to fabricate high aspect ratio features and the process is time-consuming [96].

**Figure 22:**
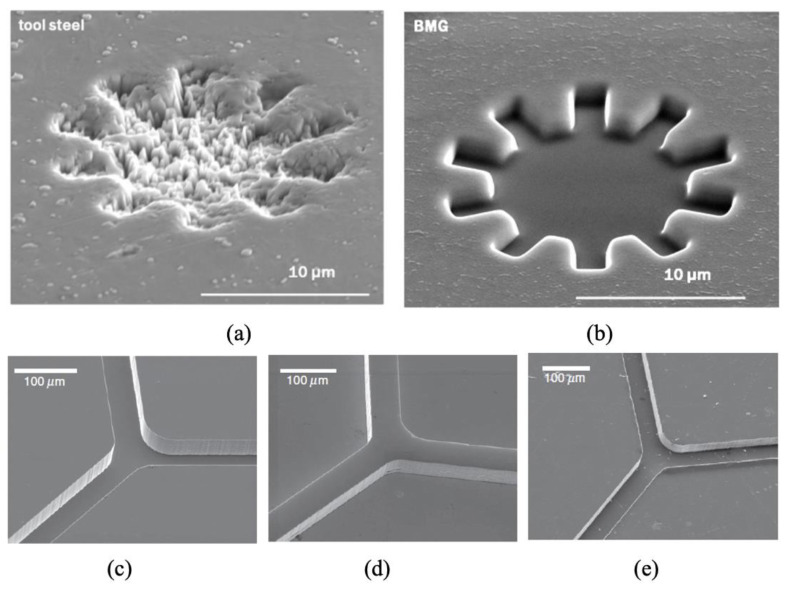
(**a**) Comparison of focused ion beam (FIB) milled patterns on conventional tool steel, (**b**) bulk metallic glass [99], (**c**) silicon master, (**d**) reversed metallic features, and (**e**) PMMA embossed patterns from metallic glass [100].

Metallic glasses, also called amorphous metals, are alloys without any intrinsic crystalline microstructure and can be patterned with features that are smaller than micron size. They also have high compressive strength, high hardness and wear resistance [99]. Figure 22 shows the Focused Ion Beam (FIB) milled features on both tool steel and bulk metallic glass. Crystals of tool steel prevented the patterning of features smaller than 10 µm (Figure 22a), whereas bulk metallic glass was easily patterned into the nanometre scale (Figure 22b). Additionally, when metallic glass is heated into its supercooling region above the glass transition temperature, it can be isothermally formed to produce multi-scale patterns. A negative channel can be patterned onto a silicon master (Figure 22c); this can then be transferred to metallic glass by hot embossing (Figure 22d); metallic glass can then be used as a tool to hot emboss PMMA (Figure 22e).

**Figure 23:**
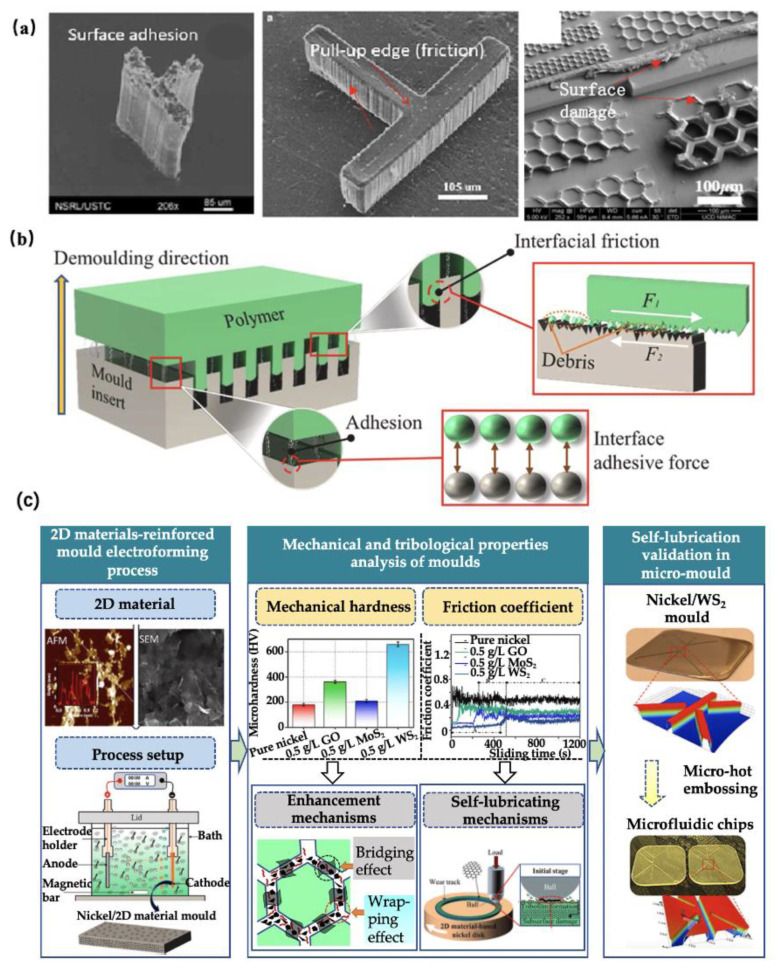
(**a**) Microinjection moulded polymeric micro structures deformation and damage, (**b**) the causes of such deformation and damage, and (**c**) 2D-reinforced high-hardness and self-lubricating nickel mould tools using nanocomposite electroforming process [101].

Nowadays, with the rapid development of advanced manufacturing and application technologies, the features on polymeric devices are increasingly miniatured, which is generally in the range of tens of micrometres or even nanometric scale. In this regard, a high-precision and high-performance mould insert is in great demand. A nickel mould insert fabricated via lithography, etching, and electroforming processes presents excellent benefits in microinjection moulding of micro/nano scale features due to its high-replication accuracy and surface finish. However, there still exists some significant technical challenges, which refer to demoulding deformation and damage of polymeric features and a limited service life of the nickel mould insert. Demoulding distortion and damage of polymeric features are due to the friction and adhesion between the sidewalls of the polymer and the mould insert during the demoulding stage of the microinjection moulding process, as shown in Figure 23a,b. In addition, nickel is a kind of soft material in comparison to tool steel, which needs to be replaced with a new one after 10,000 moulding cycles. Surface coatings technologies, such as molecular coatings, gas-phase lubricants, and liquid lubricants, have been widely used to solve the above problems. However, the coatings on the surface of the mould insert are easily worn off or introduce some contaminations into polymeric devices. Therefore, developing a high-hardness and self-lubricating mould insert by a one-step electroforming process is a critical strategy for the production of high-quality polymeric micro/nano features at a low cost. Zhang et al. [101] first proposed a 2D material-reinforced, self-lubricating nickel mould tools using nanocomposite electroforming process. The results demonstrated that 2D self-lubricating nanomaterials, such as graphene, graphene oxide, MoS_2_, and WS_2_, not only can significantly reduce the friction coefficient of mould tools, but also increased the mechanical strength of mould tools, as shown in Figure 23c. Finally, the developed nickel/WS_2_ composite mould tool was validated for the production of damage-free polymeric microfluidic chips.

### 2.4. Replication of High Aspect Ratio and Submicron Scale Features

**Figure 24:**
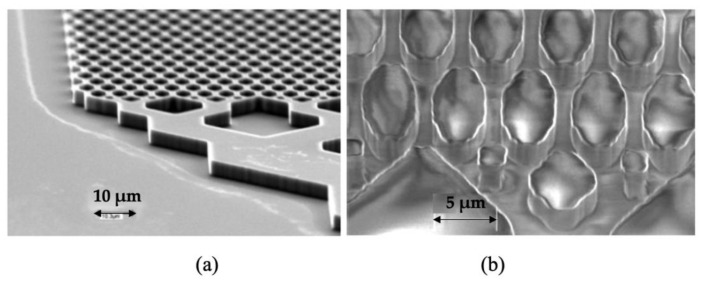
Electrochromatography microchip: (**a**) inlet and the separation column on silicon imprint master (5.1 µm in height) and (**b**) imprinted features on COC substrate [102].

Separation and mixing of fluids are common operations in chemical and biological assays. When scaling down to microfluidics, these operations are usually achieved by micropillar arrays. Figure 24 shows an electrochromatography pattern, formed by imprinting a COC substrate using a silicon master. The aspect ratio of the individual pattern and spacing are 1.5 and 2.5 [102]. Changing the surface energy by patterning the surface with high aspect ratio features has also been widely used to functionalize the polymer surface for cell or bacteria culture. However, high aspect ratio features are inclined to solidify before the cavity is fully filled. This is similar to the frozen layer problem in thin wall injection moulding. Because of limits in machine capability and material processability, the injection speed and pressure that are required over such a short cooling time are difficult to achieve in practice. When polymer melt is injected into a cavity with various thickness features, it tends to fill thicker and less resistant areas. Free-standing pillar arrays are typical features on a thick substrate. Consequently, flow hesitates at the entrance of micro features until a much thicker substrate is fully filled. The resulting hesitation time is longer than the critical cooling time of micro features, and the polymer tends to solidify at the stagnated point. A variotherm system can significantly reduce polymer viscosity and makes the filling of such features easier. Aspect ratios of up to 10 with 40 µm thick features have been achieved by the microinjection moulding process with the use of a variotherm mould heating system. Recent developments of new concepts for rapid heating and cooling of injection moulds can be also found in an excellent review [103,104].

Demoulding can easily damage high aspect ratio features either in microinjection moulding or hot embossing, especially for features without a draft angle made by lithography processes [101]. Thermal stresses may be more severe for injection moulding than for hot embossing. Thus, the demoulding temperature is probably a key factor and is associated with polymer processing and material characteristics. Experimental trials can provide an efficient way to determine a suitable demoulding process window in order to avoid feature distortion and substrate warpage. Demoulding issues and troubleshooting solutions can be found in a recent review article [105].

## 3. Application Cases of Microinjection Moulded Polymeric Devices

### 3.1. Drug Delivery

Microneedles (MNs) are comprised of many micro-projections with a wide range of geometrical designs, including different sizes in height generally from 25 µm to 2000 µm, and different shapes (solid, hollow, sharp, or flat). The MNs biological-membranes medical device has demonstrated the potential applications in drug and gene delivery by creating more molecular transportation pathways at the micro scale or even nano scale, such as the delivery of DNA into the cell [106]. The primary working principle of MNs is to penetrate the skin and directly puncture into the viable epidermis, preventing the touching of nerves and blood vessels. Therefore, the leading benefit of utilising MNs is to achieve pain-free delivery of drugs and offer better manoeuvrability of drug delivery [107]. Devoted to next-generation therapeutics, a great number of medical companies and academic communities are actively participating in the research and development of MNs.

**Figure 25:**
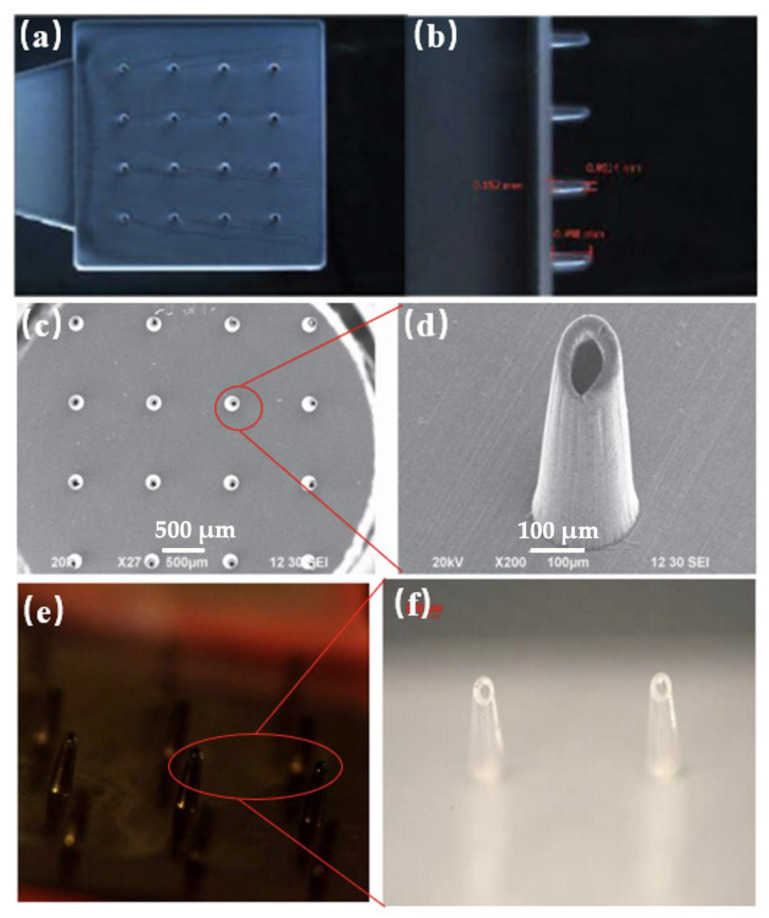
(**a**,**b**) 3D optical images of MNs profiles; (**c**,**d**) SEM images of MNs; (**e**,**f**) MNs with a droplet on the tip [112].

Extensive works have been conducted in the literature regarding the fabrication of MNs based on various technologies. With the rapid development of the MEMS (Microelectronics and Microsystems) technique, the microfabrication of MNs devices becomes possible. The first invention of MNs is achieved using silicon, however, the utility of other materials has also been progressively developed in the past decades, such as ceramic, glass, stainless steel, and functional polymers [108]. The MNs made of silicon are generally fabricated by chemical isotropic etching or reactive ion etching. The MNs with ceramic, glass, and stainless steel can be manufactured by a combination of surface and bulk micromachining or laser drilling. However, for polymer-based MNs, the principal process consists of lithography-electroforming-replication. Polymeric MNs are increasingly attracting interest from researchers, due to the extraordinary performance of polymer materials, including biocompatibility, biodegradability, good flexibility, and enhanced mechanical strength, as well as mass-production capability [109]. Microinjection moulding has been considered as a cost-effective and promising technique to fabricate polymeric MNs. The main polymeric material for MNs fabrication can be poly (methylmetha-acrylate) (PMMA), cyclic-olefin copolymer (COC), poly-L-lactic acid (PLA), poly-glycolic acid (PGA), polycaprolactone (PCL), poly-lactic-co-glycolic acid (PLGA), sodium carboxymethyl cellulose, poly (vinyl pyrrolidone), etc [110]. Sammoura et al. [111] prepared polymeric (COC) MNs using microinjection moulding, where MNs devices featured open-channel structures with the cross-sectional region of 100 × 100 µm at the top of the shank and round-shape tip having a radius of 125 µm. The fabricated MNs were verified by injecting them into a chicken leg and a beef liver, showing ~0.04 µL of liquid drawn out instantly. Yung et al. [112] fabricated sharp-tipped hollow plastic MNs via the microinjection moulding process using a mould insert with a low surface roughness. This was achieved by a picosecond laser machine, as shown in Figure 25. The mechanical strength of the MNs was analysed by combining simulation and penetration experiments. Findings showed that the MNs have sufficient stiffness and toughness that allow them to easily pierce into the skin without any breakage and distortion observed, and drugs can be successfully delivered into tissues.

### 3.2. Medical Implants

The development of engineering technologies significantly promotes the advancement of biomedical devices, especially in the field of medical implants. Main implants cover artificial organs/joints, heart valves, biosensors, stents, and scaffolds for tissue engineering [113]. The physicochemical properties of implantable materials have a great effect on the service life and practicability of implants, wherever in vitro and in vivo. Importantly, the materials of implants require sufficient mechanical strength, biocompatibility, biodegradability, and enabled cell adhesion and proliferation, etc., depending on the specific applications [114]. Advanced application of implants has extended the boundary of existing materials properties and accelerated the development of new sequence-specific materials. Among these, polymeric implants present a long-acting effect on guaranteeing patient compliance and targeting effect. For example, the PLGA/PLA has better controllability on the initial burst and release efficiency of methotrexate and the expanding and degradation of the implants [115].

**Figure 26:**
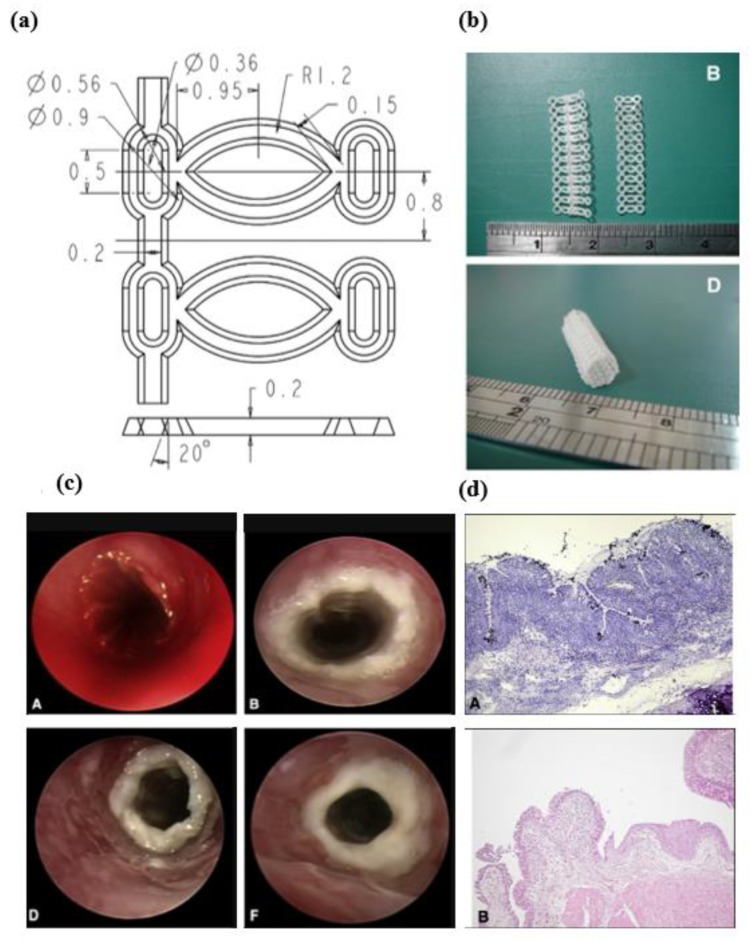
(**a**) Dimensions of the designed stent, (**b**) fabricated PCL stent, (**c**) A, immediate postoperative bronchoscopic images showed blood in the trachea; B, 1 week after surgery; D, 4 weeks after surgery; and F, 12 weeks after surgery, and (**d**) A, at 10 weeks after surgery, histological examination showed marked leukocyte infiltration in the submucosa of the stented area; B, submucosa leukocyte infiltration at 28 weeks after surgery [105].

Currently, the expandable stent implants are attracting great attention from scientific researchers and related medical firms. The stents are commonly used as medical devices in cardiac intervention operations, which have the function of dredging arteries by holding open a passageway [116]. Incorporating polymers material in stent fabrication is a growing trend due to their potential drug-holding capacity, biocompatibility, and biodegradability [117]. Therefore, the stents with a metallic scaffold-like framework provide the leading mechanical support and polymer-coated surface servicing as a biocompatible layer, and if required, a drug-loading layer. Now, the attention of researchers is gradually turning to developing purely polymeric biodegradable stent implants made of PLLA, which brings the potential convenience in spinal surgery and arterial occlusive diseases, etc. [118]. Concerning the manufacturing approaches of polymeric stents, the conventional processing method is the laser-cutting technique. Clarke et al. [119] in 2008 for the first time proposed a process route of fabricating polymeric stents using microinjection moulding. In fact, there are many apparent advantages to utilizing the microinjection mould to make a stent, such as high production efficiency, low cost, accurate reproductivity, cost-efficient tooling, and complex-structure manufacturability. Admittedly, microinjection moulding also has some common limitations in stent fabrication, such as difficulties in melt flow, cavity fully filled, and demoulding damage, etc. These will affect the surface quality of the replicated stent. As such, the related new process development and process optimisation of microinjection moulding parameters need to be focused on improving the quality of the stents. Liu et al. [120] microinjection moulded the mesh-shape bioresorbable stents made of PCL materials and validated their biocompatibility by surgically implanting them into a rabbit trachea (Figure 26). The mechanical strength of the fabricated stents was also studied. The results demonstrated integrated ciliated epithelium and remarkable leukocyte infiltration in the submucosa of the stents after 10 and 28 weeks of examination (Figure 26d). Despite the degradation rate being low, the mechanical performance was still qualified after 33 weeks.

### 3.3. Microfluidic Devices

Microfluidics is growing into a huge marker potential to many advanced applications that bridge multidisciplinary fields intersecting chemistry, physics, biology, medicine, and engineering technologies [121,122,123,124]. Microfluidic devices can be used to perform many detections and analyses such as cell separation, mixing, reaction, molecular detection, drug synthesis, and other bio-aspects. Such a microfluidic device is a so-called lab-on-a-chip that is a highly integrated system consisting of transport zones, mixing and separating zones, reaction and detection zones, and storage and waste zones [125]. Available materials for manufacturing microfluidic devices can be glass, silicon, and polymers, depending on specific manufacturing technology and applications. Currently, biomedical enterprises have motivated the rapid development of polymer microfluidic devices, benefiting from their characteristics of large-volume production capacity, low cost, good optical transparency, broad material selection range, accurate repeatability, and extraordinary biocompatibility [126]. Microinjection moulding is a commonly used technology for the mass production of polymeric microfluidic devices in a high-efficiency, cost-effective, high-precision manner [122]. These advantages are vitally important for biomedical chip applications, the majority of which are for molecular diagnostics and in which chips should be used as disposable devices for preventing any cross-infection.

**Figure 27:**
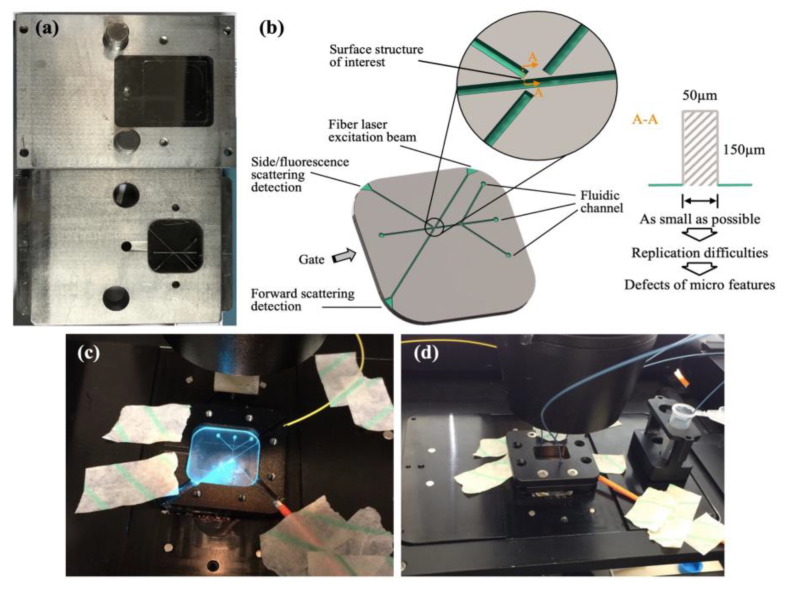
Flow cytometer chip: (**a**) cassette mould, (**b**) three-dimensional model of surface structures on the chip, (**c**) actual chip assembly, and (**d**) connection with tubing [127].

**Figure 28:**
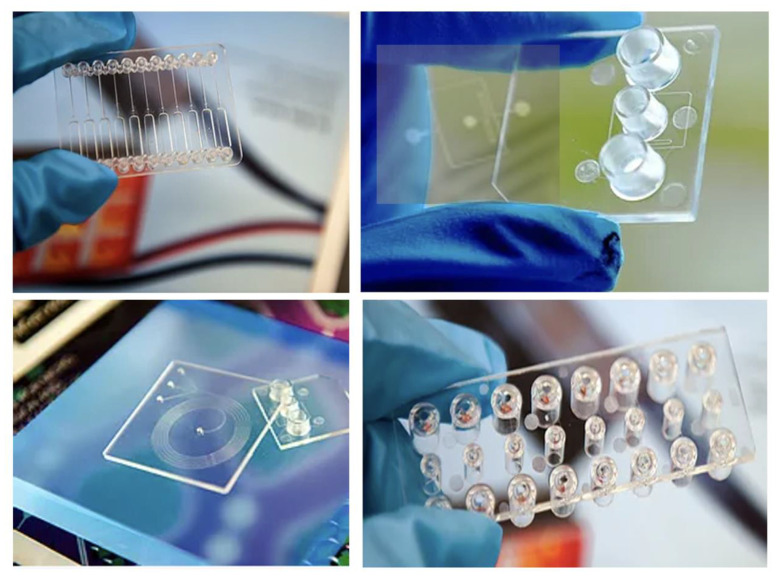
Microinjection moulded microfluidic chips in MNMT-Dublin lab.

Zhang et al. [127] microinjection moulded a flow cytometer chip with a high aspect ratio of 3 (Figure 27), where an electroformed nickel mould insert was developed. Figure 27c demonstrated a microfluidic chip bonded using a COC plate, which was fixed at a pre-designed chip holder and connected using tubing for flow testing (Figure 27d). Figure 28 shows some typical polymeric microfluidic chips fabricated in the MNMT-Dublin lab using microinjection moulding technology.

### 3.4. Functional Micro/Nano Structured Surfaces

**Figure 29:**
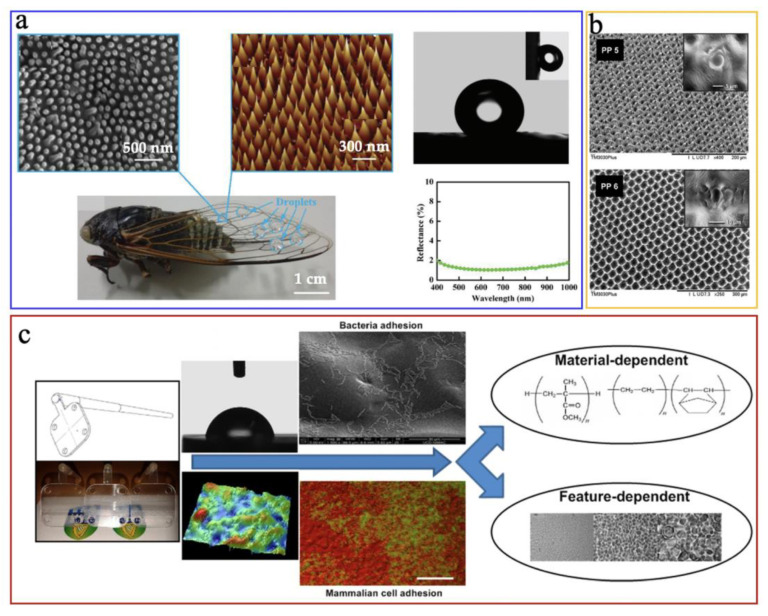
Applications of microinjection moulded functional micro/nano structured surfaces: (**a**) antireflective surfaces [127], (**b**) hydrophobic surfaces [128], (**c**) cell-adhesion surface [131].

Functional micro/nano structured surfaces can be defined as the production of the device with micro/nano scale features on its surface. The main applications of those micro/nano structured surfaces include hydrophobic/hydrophilic surfaces, self-cleaning surfaces, antibacterial surfaces, bioinspired antireflective surfaces, and cell culture surfaces, etc [128]. Microinjection moulding has been a principal technique in achieving the mass production of polymeric devices with micro/nano features. Xie et al. [129] successfully microinjection moulded the functional nano structure of the cicada wing onto polystyrene (PS) surfaces, where the PS surfaces showed a water contact angle of 143° and reflectance of ~4%, indicating an outstanding hydrophobicity and antireflectivity, as shown in Figure 29a. Romano et al. [130] microinjection moulded the textured surfaces using polypropylene(PP) discs replicated from a steel insert (shown in Figure 29b) the results showed a high-precision replication accuracy of micro features and generated the hydrophobic surfaces. Choi et al. [131] investigated the effect of micro/nano textured surfaces fabricated by microinjection moulding on cell adhesion behaviours. It was found that various feature sizes of those micro/nano textured surfaces can produce different cell adhesion effects, as shown in Figure 29c. The results indicated that developing micro/nano textured surfaces using the microinjection moulding process was a promising method to tune the cell adhesion on different areas on polymeric medical devices or implants.

### 3.5. Micro Optics

**Figure 30:**
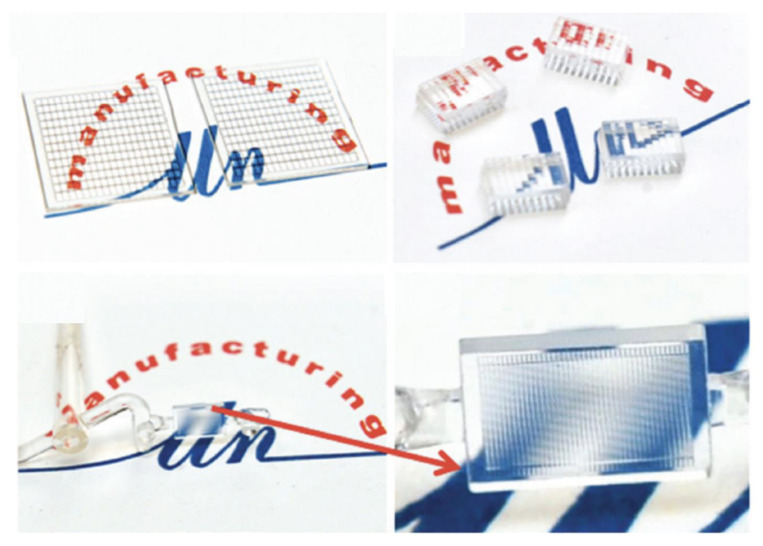
Microinjection moulded micro lenes array [133].

Polymeric optical components present many excellent advantages over glass optics in aspects of weight, manufacturability of complex surface forms, and ease of integration with other optical systems. The main applications of polymeric optics include imaging, illumination, and concentration, depending on the complexity of surface forms which could be conventional plane surfaces, spherical/aspheric surfaces, and freeform surfaces, as shown in Figure 30. Offering high-precision replication and mass-production capacities, microinjection moulding has been identified as the most efficient manufacturing technology for polymeric optics with complex geometrical features. However, high-performance polymeric optics pose harsher requirements to the microinjection moulding process relevant to the form accuracy, residual stress, and transparency control of the polymer. There are a great number of studies done in recent years on microinjection moulding of polymeric optics. The research content mainly focuses on form accuracy, residual stress, and imaging quality. Zhang et al. [132] used variotherm-assisted microinjection moulding to fabricate microlens array so as to achieve satisfactory form accuracy, surface quality, and stress birefringence of the micro lenses. The findings demonstrated that the residual stress and uniformity of the microlens array were improved by 5.08% and 88.11%, respectively, in comparison to a conventional microinjection moulding process. Futural work in microinjection moulding of polymeric optics should be concentrated on the study of optical performance and manufacturing efficiency of freeform optics.

## 4. Conclusions and Challenges

This review summarises the state-of-the-art advancement of microinjection moulding for polymeric micro devices and their typical applications. The recent decade has witnessed major developments in the technology that made it one of the most preferred high-volume techniques for fabricating polymeric micro devices. The expanded applications of polymeric micro devices and surface features inspire research on microinjection moulding from various perspectives: machine and mould, process measurements and optimisation, numerical simulation, rheology, morphology, properties studies, and advanced applications. Overall, the fundamental development of microinjection moulding for polymeric micro devices has generated novel machines, a large amount of process knowledge, and sufficient process know-how for novel product development.

A number of limitations, however, need to be overcome before the mass-production of polymeric micro devices. Mould design is important for the replication of high-quality features and auxiliary equipment, such as a variotherm mould temperature controlling system and a vacuum venting system, are required. Semiconductor fabrication technologies enable the fabrication of larger substrates with micro/nano scale features. Multi scale integration of such features on a metallic insert is still rare. In-line functionality integration, such as electrodes, lamination, bonding, and packaging are urgently needed to produce commercial microfluidics devices. Studying typical micro/nano features on a large substrate, and exploring a routine for process optimisation and quality control for both micro devices and micro/nano features, have important practical uses and value. Development of the entire process chain, including microinjection moulding for advanced applications, would have great potential. Integrated micro scale production based on industry 4.0 of micro injection moulding for polymeric micro parts and micro/nano surface structures will be important for high precision scale-up production of complex polymeric micro/nano products.

## Figures and Tables

**Table 1:** List of microinjection moulding machines commercially available and their characteristics [12].

Manufacturer	Model	Clamp Force (kN)	Injection Capacity (cm^3^)	Injection Pressure (Bars)	Plasticization (Screw or Plunger)	Injection Speed (mm s^−1^)
Lawton (Fridingen, Germany)	Sesame Nanomolder	13.6	0.082	3500	10 mm plunger	1200
APM (Taichung, Japan)	SM-5EJ	50	1	2450	14 mm screw	800
Battenfeld (Barcelona, Spain)	Microsystem 50	56	1.1	2500	14 mm screw	760
Nissei (New Taipei, Japan)	AU3	30	3.1		14 mm screw	
Babyplast (Lyon, France)	Babyplast 6/10	62.5	4	2650	10 mm plunger	
Sodick (Warwick, UK)	TR05EH	49	4.5	1970	14 mm screw	300
Rondol (Nancy, France)	High Force 5	50	4.5	1600	20 mm screw	
Boy (Exton, PA, United States)	12/AM	129	4.5	2450	12 mm screw	
Toshiba (Troy, MI, United States)	EC5-01.A	50	6	2000	14 mm screw	150
Fanuc (Yamanashi, Japan)	Roboshot S2000-I 5A	50	6	2000	14 mm screw	300
Sumimoto (Suwanee, GA, United States)	SE7M	69	6.2	1960	14 mm screw	300
Milacron (Batavia, NY, United States)	Si-B 17 A	147	6.2	2452	14 mm screw	
MCP (America North (USA-Canada-Mexico))	12/90 HSE	90	7	1728	16 mm screw	100
Nissei (New Taipei, Japan)	EP5 Real Mini	49	8	1960	16 mm screw	250
Toshiba (Michigan, United States)	NP7	69	10	2270	16 mm screw	180

**Table 2:** Comparison of micro moulds and inserts manufacturing techniques [86].

Technology	Minimum Feature Size (µm)	Surface Roughness (µm)	Aspect Ratio	Material	Manufacturing Cost ($)	Other Applications
Micro milling	25–100	0.2–5	10	Brass, COC, Steel	500~1000	Microstructures and micro-texturing in MEMS devices, micro-fuel cells, microfluidic chips, EDM electrodes, and optics, etc.
Micro electro discharge machining	10–25	0.05–1	50–100	Conductive material	~3000	Inkjet nozzles of printers, cooling holes of turbine blades, and honeycomb structures, etc.
Micro laser machining	1–5	0.4–1	<50	Any	~3000	Photonics, surface plasma resonance, optoelectronics, bio-sensing, micro/nanofluidic, etc.
Micro electrochemical machining	10	0.02	NA	Conductive material	NA	Turbine blades, shaving heads, artillery projectiles, and surgical implants, etc.
X-ray lithography	0.5	0.02	100	Photoresist	>10,000	Diffractive and refractive optics, spectrometer, X-ray grating interferometry, and mask, etc.
Ultraviolet lithography	0.7–1.5	NA	22	Photoresist	1000	
Deep reactive ion etching	2	NA	10-20	Silicon	1000~3000	MEMS devices, memory circuits, mask, and flexible electronics, etc.
Focused ion beam lithography	0.1	NA	3	Any	1000~5000	Semiconductor devices, integrated circuits, bio-sensing, and nano-optics, etc.
Electroforming	0.3	0.1	<10	Conductive material	1000~3000	CDs, DVDs, Blu-ray discs, metal mesh, micro-optics, microfluidics, and microelectronics, etc.
